# 3DoF+ 360 Video Location-Based Asymmetric Down-Sampling for View Synthesis to Immersive VR Video Streaming

**DOI:** 10.3390/s18093148

**Published:** 2018-09-18

**Authors:** JongBeom Jeong, Dongmin Jang, Jangwoo Son, Eun-Seok Ryu

**Affiliations:** Department of Computer Engineering, Gachon University, Seongnam 13120, Korea; uof4949@gc.gachon.ac.kr (J.J.); dogzz9445@gc.gachon.ac.kr (D.J.); sjw6757@gc.gachon.ac.kr (J.S.)

**Keywords:** virtual reality, 3DoF+, HEVC, view synthesis, VSRS, multi-view video coding

## Abstract

Recently, with the increasing demand for virtual reality (VR), experiencing immersive contents with VR has become easier. However, a tremendous amount of calculation and bandwidth is required when processing 360 videos. Moreover, additional information such as the depth of the video is required to enjoy stereoscopic 360 contents. Therefore, this paper proposes an efficient method of streaming high-quality 360 videos. To reduce the bandwidth when streaming and synthesizing the 3DoF+ 360 videos, which supports limited movements of the user, a proper down-sampling ratio and quantization parameter are offered from the analysis of the graph between bitrate and peak signal-to-noise ratio. High-efficiency video coding (HEVC) is used to encode and decode the 360 videos, and the view synthesizer produces the video of intermediate view, providing the user with an immersive experience.

## 1. Introduction

As the virtual reality (VR) market is expanding rapidly, the need for efficient immersive VR technology has become more important. To play high-quality VR video through a head-mounted display (HMD), the minimum resolution of the video must be 4K. In this case, the amount of data to be processed from the HMD increases rapidly. Therefore, the Moving Picture Experts Group (MPEG) proposed a technology, which processes the viewport of the user, called motion-constrained tile set (MCTS) [1] in 2016; further, a paper describing MCTS implementation for VR streaming was submitted [2]. Moreover, to provide the user with high-quality 360 videos, region-wise packing [3] was proposed. It encodes a region of interest (ROI) with high quality and the other regions with low quality.

To support immersive media, the MPEG-I group, established by MPEG, divided the standardization associated with VR into three phases, namely three degrees of freedom (3DoF), three degrees of freedom plus (3DoF+), and six degrees of freedom (6DoF) [4]. In 3DoF+ and 6DoF, multi-view 360 videos are required and they comprise texture and depth images to support 3D video [5]. Because both the phases provide 360 videos in response to a user’s movement, it is inevitable to synthesize the immediate views using existing views. View Synthesis Reference Software (VSRS) for 360 videos [6], Reference View Synthesizer (RVS) [7], and weighted-to-spherically-uniform peak signal-to-noise ratio (WS-PSNR) for 360 video quality evaluation [8] were proposed to MPEG to create virtual views and evaluate them.

**Figure 1:**
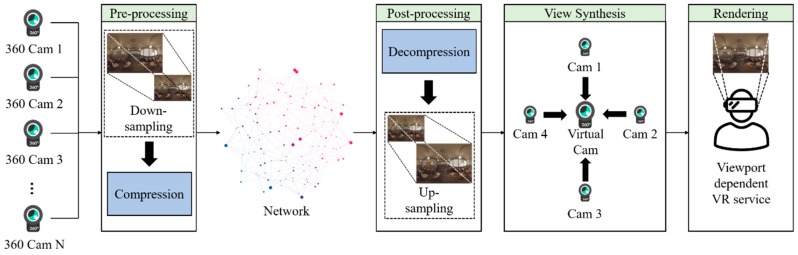
Viewport-dependent immersive VR service with VLADVS.

A large amount of bandwidth is required for transmitting 3DoF+ or 6DoF 360 videos because such videos need both high-resolution texture and depth. To overcome this problem, down-sampling or region-wise packing could be applied. In this paper, we propose the View Location-based Asymmetric Down-sampling for Vie Synthesis (VLADVS) concept for the bitrate decreasing system with appropriate down-sampling ratio and a quantization parameter for 3DoF+ texture and depth in view synthesis, as shown in Figure 1. It introduces a pilot test with Super Multiview Video (SMV) [9] and 3DoF+ test sequences. Finally, it provides the rate distortion (RD) curve of bitrate and WS-PSNR obtained by 3DoF+ test sequences using 360lib with HEVC.

This paper is organized as follows: Section 2 introduces about related work such as the MPEG 360 video standard, multi-view video coding, and view synthesis. Section 3 explains the overall experiment, including view synthesis with free viewpoint television (FTV) test sequences and 3DoF+ video test sequences. Section 4 summarizes the result of the experiment for proposed system. Lastly, Section 5 presents our conclusions and future work.

## 2. Related Work

### 2.1. 360 Video Standard in MPEG

**Figure 2:**
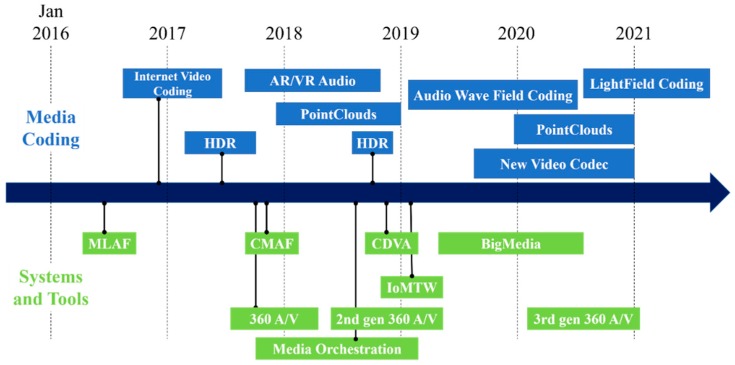
MPEG standardization roadmap.

During the 116th MPEG meeting, the MPEG-I group was established for the support of immersive media. They began work by standardizing the format of immersive, omnidirectional video in 2017 [10]. Figure 2 shows the standardization roadmap of MPEG. MPEG-I group divided the standardization into three phases [11]. Phase 1a aims to provide 360 video and contents including stitching, projection, and video encoding.

**Figure 3:**
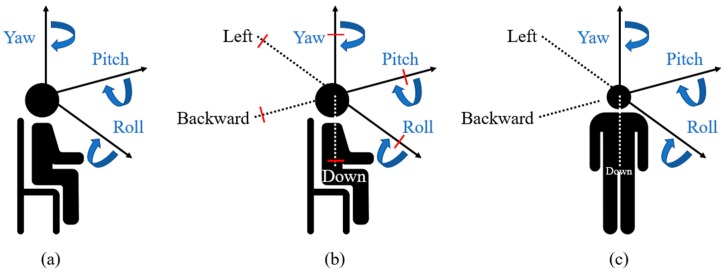
Viewing angle and the degree of freedom: (**a**) 3DoF; (**b**) 3DoF+; and (**c**) 6DoF.

Figure 3 introduces the 3DoF, 3DoF+, and 6DoF viewing angle and degree of freedom. If a user watches the stereoscopic video, the movement of the user is defined along the three directions, namely yaw, pitch, and roll. However, in the 3DoF video, the things behind the objects cannot be represented, indicating the limited experience of VR.

**Figure 4:**
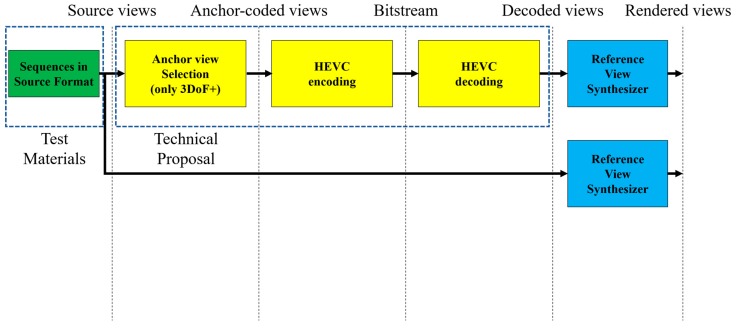
Anchor generation structure of 3DoF+.

To overcome the limitations of 3DoF, the concept of 3DoF+, part of phase 1b in MPEG-I, was proposed. 3DoF+ provides limited movements of yaw, pitch, and roll, as described in Figure 4. Thus, it provides more immersive experience than 3DoF.

In 3DoF+, the VR device must offer the video of view that the user watches. If this video of view is not included in the original video, 3DoF+ system synthesizes the view that did not exist before. Thus, Reference Intermediate View Synthesizer [12] is required. Further, to synthesize virtual views, additional depth information, such as distances between camera and objects, must be supplied. As it requires a large amount of data to be transmitted, optimization for data transmission and compression must be proposed.

As the solutions to the abovementioned problems, enhanced communication technologies such as 5G mobile technology [13] and mobile data offloading [14] have been announced recently. Moreover, the amount of resources used by the video transmission system is limited in a mobile platform. Since the limited resource is a weakness to the mobile device, some solutions using adaptive video transmission system [15] or interactive media system [16] were proposed. Considering the structure of CPU in a mobile device, asymmetric multicore processing [17,18] was proposed to use its resource efficiently. Furthermore, scalable video coding [19,20] or multiple layer video coding [21] can be applied as the 3DoF+ video contains multiple videos.

View synthesis assumes video transmission from the server to the client. Therefore, the video must be compressed, as shown in Figure 4. The anchor view is used in view synthesis, which should be encoded and decoded. Subsequently, phase 2 of MPEG-I deals with 6DoF, which means 3DoF+ with translational movements along the *X*-, *Y*-, and *Z*-axes. It supports the user’s movements including walking, as described in Figure 3.

### 2.2. Multi-View Video Coding

Multi-view video provides the user with an immersive 3D experience. Such video provides diverse views gained from one scene simultaneously. Particularly, 3D multi-view video includes both texture and depth information. It enables users to have multiple views of what they intend to watch. MPEG defined a 3D video system [22], which is a part of FTV, and it contains multi-view video acquisition, encoding, transmission, decoding, and display. To process the multi-view video efficiently, multi-view video coding [23,24] is required.

Multi-view videos have common features as they contain the same scene at the same time. The difference between each view is the indigenous point of view; that is, a multi-view video of one viewpoint can be made by referencing another view.

**Figure 5:**
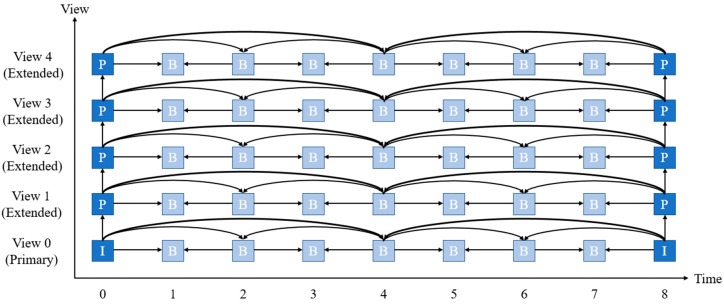
Multi-view video encoding view reference structure.

Figure 5 shows the hierarchical B frame multi-view video encoding structure between primary view and extended views. The blue box represents a key frame referenced by the B frame. The I frame can be reconstructed while the P frame is referenced by one frame. The B frame is referenced by two frames when predicting. Joint multi-view video model [25] for reference software model of multi-view video coding was proposed to compress multi-view video while containing compatibility with H.264.

### 2.3. View Synthesis

Although multi-view video provides some views, it cannot offer out-of-source views. Because multi-view video coding requires a large amount of data and computing power to process, the number of views the multi-view video can support is limited. Accordingly, view synthesis for multi-view video [26,27] was developed to overcome the limitation of multi-view video coding. When using view synthesis, the server does not need to send all the source views because it synthesizes dropped views that were not sent. Further, if the video provider did not acquire many source views due to the limitation of resources such as a camera and the amount of data, the other views not offered by the provider can still be synthesized.

**Figure 6:**
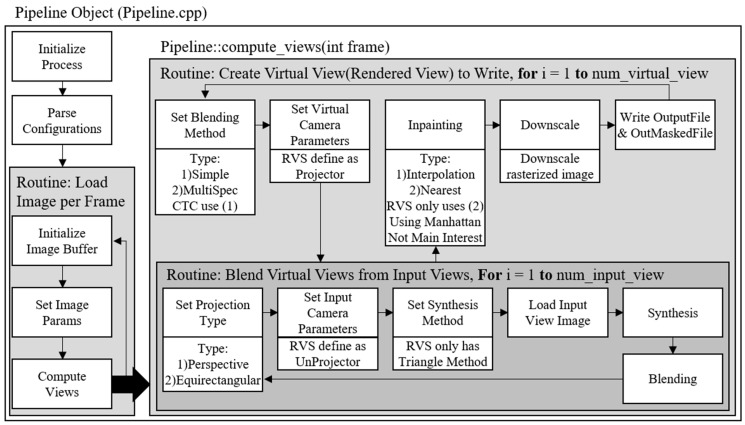
View synthesis pipeline of RVS.

Figure 6 illustrates how to synthesize the intermediate views with RVS 1.0.2 [28]. It requires a texture video, depth map, and camera parameter. Depth map [29,30] represents the distance between the camera and the object shown in the texture video. The purpose of the depth map is to represent a 3D space, which is also used by the haptic system [31,32]. If the depth map format is 8-bit, the range of the depth value is between 0 and 255. The depth map can be obtained by a depth camera that uses a depth sensor; otherwise, it can be generated by depth estimation software. MPEG-4 group proposed Depth Estimation Reference Software [33,34] to obtain the depth map from the texture video efficiently.

**Figure 7:**
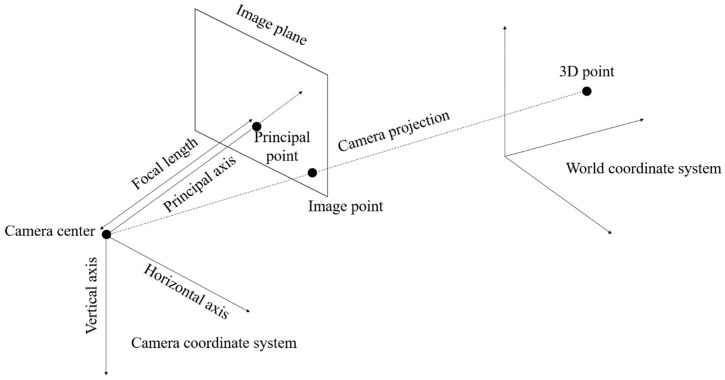
Image projection in a pinhole camera.

**Figure 8:**
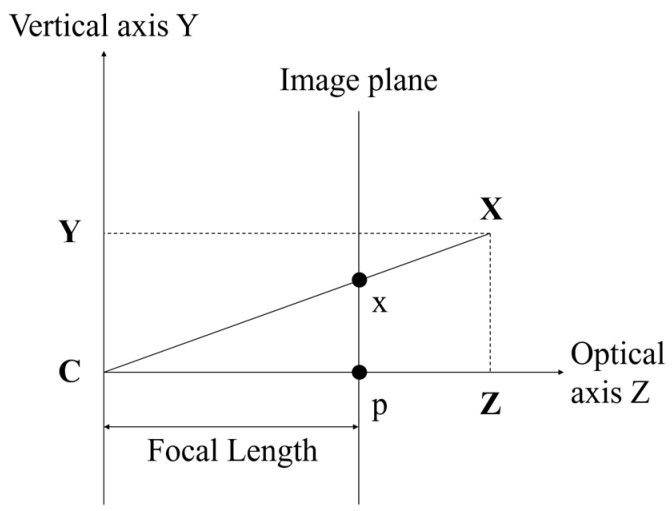
Image plane in camera coordinate system.

Generally, the multi-view video is obtained from a pinhole camera. It projects the actual object onto a 2D plane image, as shown in Figure 7. The projection is implemented using a world coordinate system and camera coordinate system. The world coordinate system presents a 3D space. The camera is located in the world coordinate system, and it also has a 3D coordinate system. The center point of the camera represents the location of the camera in the world coordinate system. The camera coordinate system has *X*-, *Y*-, and *Z*-axes. The *X*-, *Y*-, and *Z*-axes represent the horizontal, vertical, and optical axis (also called principal axis), respectively. The optical axis is the direction of the camera ray. The principal point is the intersection point between the principal axis and the image plane. The distance from the camera center to the principal is called focal length, as shown in Figure 8. Each point of the object in the 3D space is projected onto a 2D image plane by the camera.

To obtain the intermediate view, the point coordinates from reference views must be converted into the synthesized view. Each reference view, which is used to synthesize the intermediate view, has its own camera coordinate system. If we realize the camera parameter of reference views and intermediate view, the camera coordinate system of intermediate view can be generated using the world coordinate system. Once the conversion is complete, texture mapping from the reference views to intermediate view is performed.

## 3. View Location-Based Asymmetric Down-Sampling for View Synthesis

**Figure 9:**
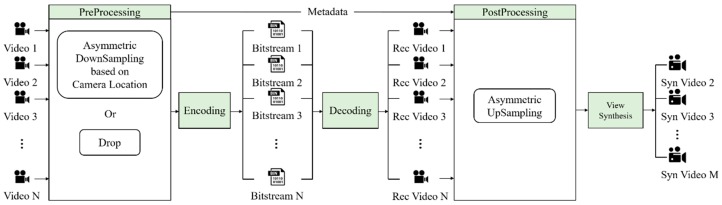
View Location-based Asymmetric Down-sampling for View Synthesis. Rec stands for reconstructed, and Syn stands for synthesized.

This section explains VLADVS for efficient use of bandwidth in video transmission, as described in Figure 9. It allocates the down-sampling ratio to the videos based on the distance between the input video and the video that needs to be synthesized. If the input video is close to the synthesized video, the proposed system assigns low down-sampling ratio because the video near the synthesized video has a great influence on the quality of synthesized video. Section 3.1 explains view synthesis with FTV multi-view test sequences to decide the down-sampling ratio. Section 3.2 presents the results of source view synthesis with 3DoF+ video test sequences, which implies the impact of the input view number and the relation of the correlation between the down-sampling ratio of texture and depth in view synthesis. Finally, Section 3.3 proposes the intermediate view synthesis method and conditions for 3DoF+ video.

### 3.1. View Synthesis with FTV Multi-View Test Sequences

To reduce the bitrate when transmitting multi-view video, this paper proposes a low-complexity multi-view video transmit system including down-sampling and up-sampling. The feasibility of this method was proved by a pilot test with FTV multi-view sequences [35].

**Figure 10:**
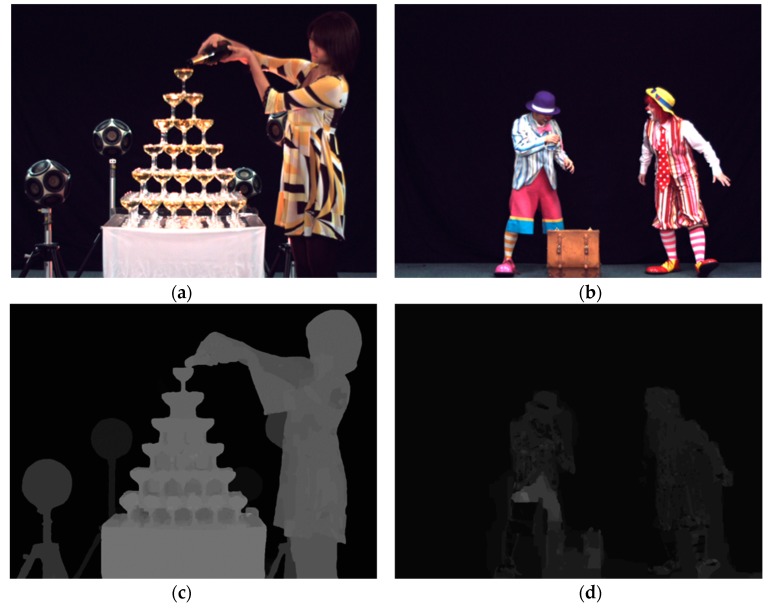
FTV test sequences from Nagoya University. (**a**) Champagne_tower (1280 × 960), obtained from 80 cameras with stereo distance, consists of 300 frames with 30 fps; (**b**) pantomime (1280 × 960), gained from 80 cameras with stereo distance, consists of 300 frames with 30 fps; (**c**) depth map of Champagne_tower; (**d**) depth map of Pantomime.

Champagne_Tower and Pantomime sequences, as shown in Figure 10, were used. The resolution and number of frames of Champagne_Tower and Pantomime sequences are 1280 × 960 (acquired from 80 cameras) and 300, respectively.

**Figure 11:**
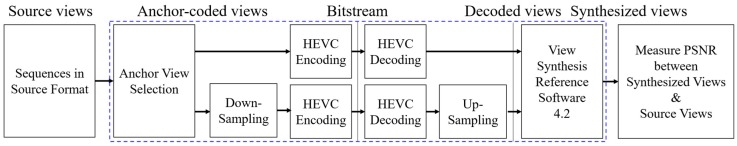
Proposed system architecture with FTV test sequences.

Figure 11 introduces the proposed system architecture with FTV multi-view test sequences. First, it selects the anchor view, i.e., the source view used to synthesize the intermediate view. Test sequences provide the depth map of 37, 39, and 41 views, i.e., anchor view which requires both texture and depth. The combinations of view synthesis are presented in Table 1. Second, it down-samples the selected anchor views. The down-sampling ratios are 0, 20, 40, 50, and 75(%), as shown in Table 2. For down-sampling and up-sampling, the DownConvertStatic executable in Joint Scalable Video Model (JSVM) [36] was used. Third, it encodes and decodes the down-sampled views. For encoding and decoding, HEVC reference software (HM) version 16.16 [37] was used. VSRS 4.2 [38] was used to synthesize the intermediate view. Fourth, it up-samples the decoded views. Fifth, it synthesizes the intermediate view by referencing up-sampled anchor views. Finally, it measures the PSNR between the original intermediate views and synthesized views for objective quality evaluation. For PSNR measurement, the PSNR static executable of JSVM was used.

**Figure 12:**
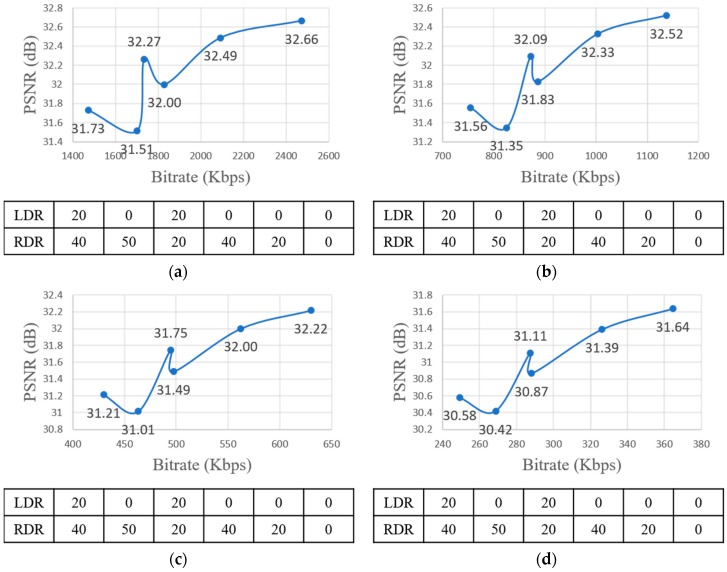
RD-curve between PSNR and average bitrate with different QPs. LDR stands for Left view Down-sampling Ratio, RDR stands for Right view Down-sampling Ratio. (**a**) RD-curve with QP = 22; (**b**) RD-curve with QP = 27; (**c**) RD-curve with QP = 32; (**d**) RD-curve with QP = 37.

**Figure 13:**
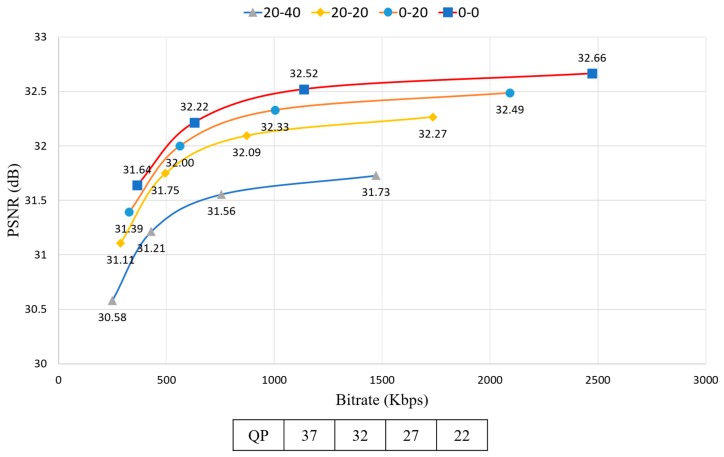
RD-curve between PSNR and average bitrate with different down-sampling ratio combinations.

For encoding, the quantization parameter (QP) values are 22, 27, 32, and 37. In a pilot test with FTV multi-view sequences, the experiment was executed for every combination of down-sampling ratio, QP, and view synthesis. The pilot test results are shown in Figure 12 and Figure 13. The figures show the RD-curve between PSNR and average bitrate with different QPs. The reason why the graph shows the combination 0-0 to 20-40 is because it only includes the combinations whose difference values with the original view combination (0-0) are under 1. Even though the average down-sampling ratio of the combination 0-40 (left view-right view) is equal to 20-20, the PSNR value of 20-20 was higher than 0-40. Moreover, the average bitrate of 20-20 was smaller than 0-40. Figure 12 implies that PSNR of the uniform down-sampling ratio assignment of left and right view is higher than non-uniform down-sampling ratio assignment. Furthermore, the performance of 20-40 was better than 0-50 because the down-sampling ratio difference value for each left and right view of 20-40 was lower than 0-50 even though the average down-sampling ratio of 20-40 was greater than 0-50.

Figure 13 shows the RD-curve between PSNR and average bitrate with different down-sampling ratio combinations. In the case of 20-20, the difference value between QP = 27 and QP = 22 is 0.17, which is very low whereas the difference value of bitrate is 862.6038, which is very high.

### 3.2. Source View Synthesis with 3DoF+ Test Sequences

**Figure 14:**
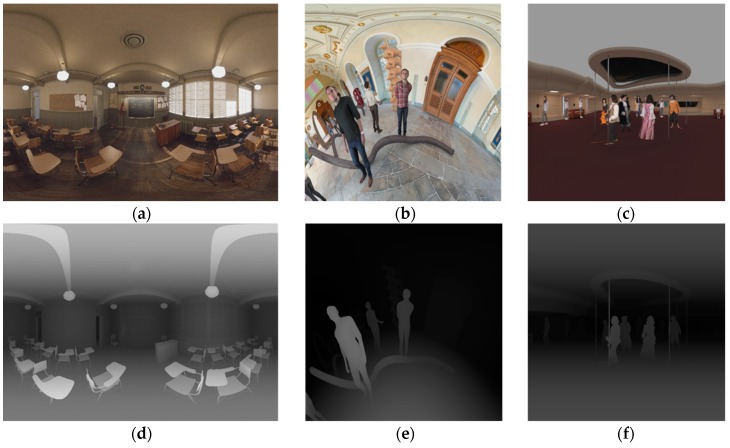
3DoF+ test sequences. (**a**) ClassroomVideo (4096 × 2048), 360° × 180° FOV ERP, consists of 15 source views, has 120 frames, 30 fps; (**b**) TechnicolorMuseum (2048 × 2048), 180° × 180° FOV ERP, consists of 24 source views, has 300 frames, 30 fps; (**c**) TechnicolorHijack (4096 × 4096), 180° × 180° FOV ERP, consists of 10 source views, has 300 frames, 30 fps; (**d**) depth map of ClassroomVideo; (**e**) depth map of TechnicolorMuseum; (**f**) depth map of TechnicolorHijack.

**Figure 15:**
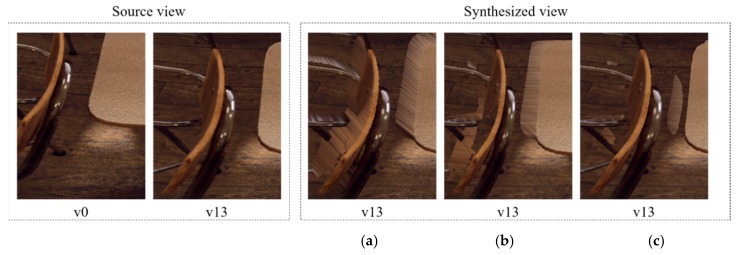
View synthesis of ClassroomVideo for subjective quality evaluation. (**a**) View v13 synthesized from view v0; (**b**) view v13 synthesized from view v0, v11; (**c**) view v13 synthesized from view v0, v11, v14.

For the 3DoF+ experiment, MPEG provides Classroom-Video [39], TechnicolorMuseum, and TechnicolorHijack as test sequences, which are illustrated in Figure 14. The pilot test was conducted on ClassroomVideo. To verify if the number of input views influences the quality in view synthesis, RVS set v0, v11, and v14 as source views, which are not encoded, and v13 for the intermediate view. Figure 15 shows the pilot test of ClassroomVideo for subjective quality evaluation. As the number of input views increased, the overlapped regions of the synthesized views decreased. That is, the subjective quality increases when RVS achieves several input views. However, the texture quality of the synthesized view decreased when the number of input views increased.

**Figure 16:**
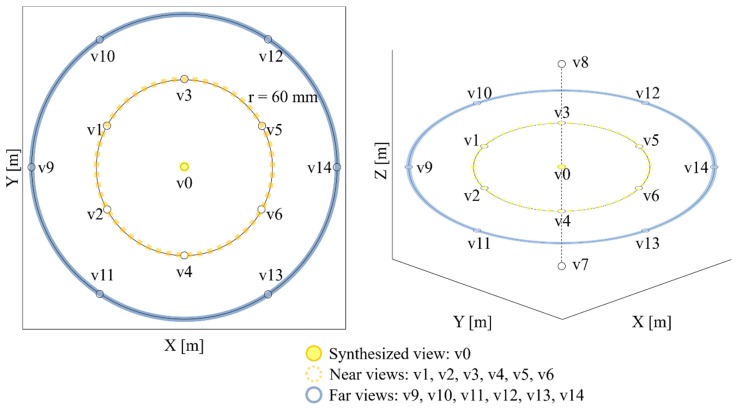
ClassroomVideo viewpoint definition for objective quality evaluation.

In another experiment, view v0 was defined as a synthesized view; v1, v2, v3, v4, v5, and v6 were called near views; and v9, v10, v11, v12, v13, v14 were called far views, as shown in Figure 16. The distances between the synthesized view and the near and far views are same. For objective quality evaluation, WS-PSNR tool [40] was used.

Table 3 shows the WS-PSNR for synthesized source views of ClassroomVideo. WS-PSNR value of (6) was higher than (1) although (6) has fewer views. Adding more views, which are down-sampled, is not appropriate for the quality of the synthesized view. If the input views were closer to the synthesized view, its PSNR value would be higher, as we can see by comparing (1) and (3). Interestingly, the PSNR value of (1) was higher than (2) although the depth maps of (2) were not down-sampled. It implies both the texture and the depth should be down-sampled with the same ratio.

### 3.3. Intermediate View Synthesis with 3DoF+ Test Sequences

**Figure 17:**
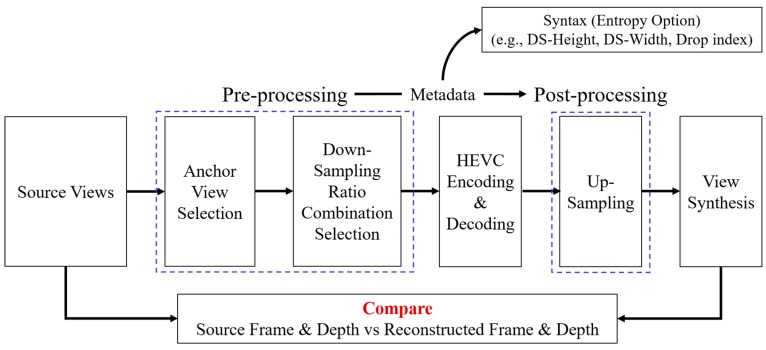
Proposed system architecture for intermediate view synthesis of 3DoF+ video.

In Section 3.2, the source view synthesis with 3DoF+ test sequences was introduced. Because the 3DoF+ common test condition (CTC) of 3DoF+ requires the ability to synthesize the intermediate views, which do not exist in source views, this section introduces the view synthesis of intermediate view. The proposed system architecture, VLADVS, includes anchor view selection, down-sampling ratio combination selection, down-sampling, encoding, decoding, up-sampling, view synthesis, and measuring WS-PSNR, as described in Figure 17.

**Figure 18:**
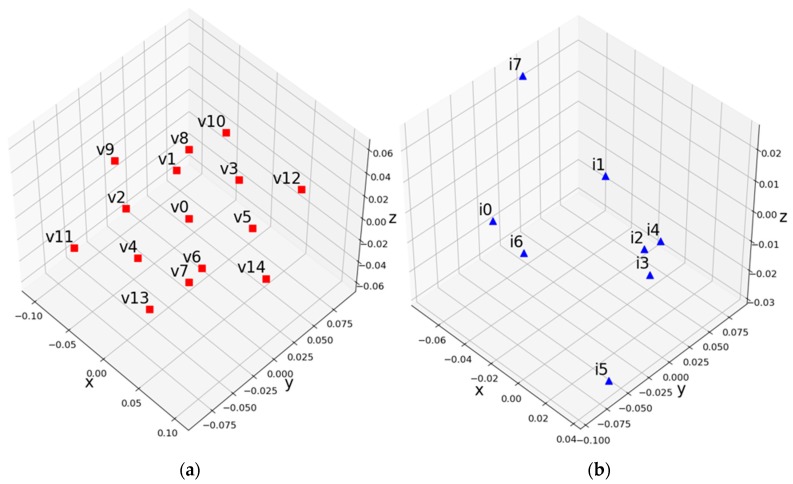
Source and intermediate view position. (**a**) Source view; (**b**) intermediate view.

In CTC, the QPs used for texture and depth are shown in Table 4. The difference value between the texture and depth QP is 5, which was decided by an experiment [41]. Table 5 shows the resolution of the down-sampling ratio for ClassroomVideo. Down-sampling is applied to both texture and depth. 360ConvertStatic of 360lib 5.1 was used for down-sampling. Table 6 shows the anchor-coded views per class or ClassroomVideo. Class A1 uses all views for view synthesis, whereas class A2 and class A3 use the subset of views. To reduce the view synthesis runtime, frame ranges for view synthesis were set in CTC as shown in Table 7. Because the proposals for 3DoF+ are required to generate ERP video for all intermediate view positions, the experiment was designed to synthesize the intermediate views using A1, A2, and A3 class views. Figure 18 shows the positions of the source and intermediate views.

The goal of this experiment is to reduce the bitrate while conserving the PSNR. Modifying parameters such as down-sampling ratio, QP, and the number of input views to optimize them are included in the experiment, which is explained in Section 4.

## 4. Experimental Results

In Section 3.3, the intermediate view synthesis was introduced. As described in Section 2.3, RVS was used for view synthesis. In addition, the tool used for down-sampling and up-sampling is 360Convert in 360lib 5.1, and for HEVC encoding and decoding, the HM 16.16 encoder and decoder are used. The used version of RVS is 1.0.2 with openCV 3.4.1, and the server used for experiment has 2 Intel Xeon E5-2687w v4 CPU and 128 GB.

Table 8 shows the summary of WS-PSNR_Y with different down-sampling ratios for regular outputs and masked outputs in synthesizing the intermediate views. It contains the WS-PSNR_Y values of synthesized intermediate views. The results of the regular output were better than the masked outputs. Further, class A2 and class A3, which discarded some source views, showed low WS-PSNR. For down-sampling the anchor views, the ratio 12.5% is reasonable. Table 9 contains WS-PSNR_Y of synthesized views for different QPs with A1 class. This shows that the difference value of WS-PSNR_Y between R1 and R2 is not high.

**Figure 19:**
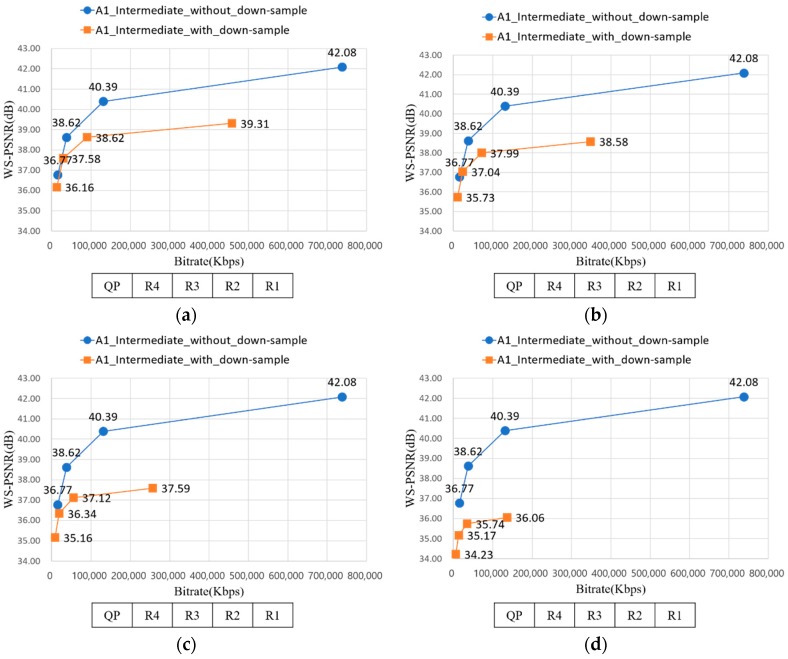
RD-curve between WS-PSNR_Y and bitrate of A1. (**a**) RD-curve with 12.5% down-sampling; (**b**) RD-curve with 25% down-sampling; (**c**) RD-curve with 37.5% down-sampling; and (**d**) RD-curve with 50% down-sampling.

Figure 19 depicts the RD-curve between WS-PSNR_Y and bitrate of A1 with 12.5%, 25%, 37.5%, and 50% down-sampling ratios. The values of the *X*-axis were QP of R1–R4. R2 can be used instead of R1; the gap between R1 and R2 was not high. With QP of R2 and 12.5% down-sampling ratio, it saved approximately 87.81% bitrate while losing only 8% WS-PSNR, compared to the result of R1 and 0% down-sampling ratio.

In addition, experiment with two down-sampling ratios was conducted. After sorting the source views by the distance between the source views and intermediate views, the experiment assigned two down-sampling ratios to the source views. If the source views are close to the intermediate view, they got low down-sampling ratios. To decide the combination of two down-sampling ratios, the following formula is used:

nCr(1)

Here, n is the number of the entire down-sampling ratios, and r is the number of the down-sampling ratios to assign. Table 10 shows the combinations of two down-sampling ratios deducted by Equation (1).

To obtain the number of DR1 and DR2 to the source views, the following equations are used:

(2) $$n\left(\mathrm{DR}1\right)=\lceil \frac{n\left(source\;views\right)}{2}\rceil$$

(3) n(DR2)=n(source views)−n(DR1) 

Equation (2) explains how to calculate the number of DR1. After dividing the number of source views with 2, which means the number of down-sampling ratios to assign, the formula rounds up the result. DR2 is set to the difference value between the number of source views and the number of DR1, as shown in Equation (3).

**Figure 20:**
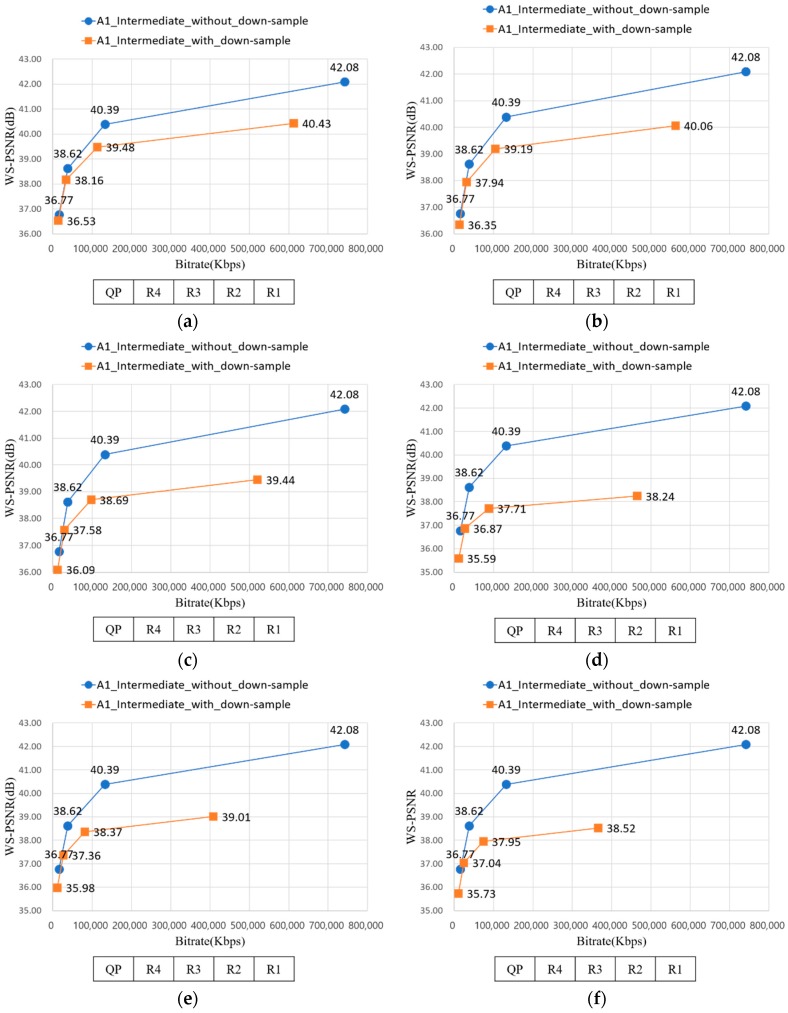

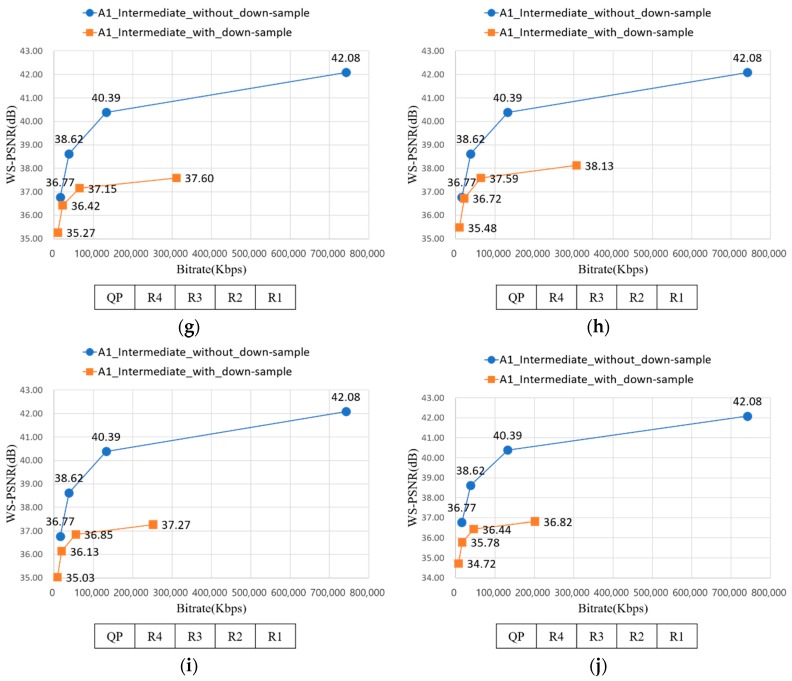
RD-curve between WS-PSNR_Y and bitrate of A1. (**a**) RD-curve with D1; (**b**) RD-curve with D2; (**c**) RD-curve with D3; (**d**) RD-curve with D4; (**e**) RD-curve with D5; (**f**) RD-curve with D6; (**g**) RD-curve with D7; (**h**) RD-curve with D8; (**i**) RD-curve with D9; and (**j**) RD-curve with D10.

Figure 20 represents the RD-curve between WS-PSNR_Y and bitrate of A1 with D1 − D10. In Section 3.1, uniform down-sampling ratio assignment showed better PSNR value than non-uniform down-sampling ratio assignment. Likewise, although the average down-sampling ratio of Figure 20d and Figure 19b are the same, but the WS-PSNR value of the latter is better. It implies the uniform down-sampling is an advantage for view synthesis.

In Section 3.3, down-sampling the source views far from the intermediate view is better in WS-PSNR value than down-sampling the near views from intermediate view. Equally, the WS-PSNR value of down-sampling the near views from intermediate views, as described from Figure 20a, is higher than Figure 19a. Although the former requires more bitrate than latter, the difference value is 23,371 Kbps when QP is R2, which is not greatly high. It implies down-sampling the far views from intermediate views can be a method for saving bitrate while preserving the WS-PSNR value.

## 5. Conclusions

This paper proposes a bitrate-reducing method for 3DoF+ video synthesis and transmission. Particularly, by down-sampling and up-sampling the texture and depth, the proposed method saves the bitrates of bitstream file while degrading the objective video quality very little in WS-PSNR. In addition, down-sampling the far views brings higher WS-PSNR value than down-sampling all the source views. However, because the number of the parameters for the experiment was not enough to deduct the optimal parameter for view synthesis, the experiment using video compression methods such as region-wise packing [42] must be conducted to reduce the bitrates for immersive 360 VR video streaming. Furthermore, intensive experiments should be carried out to derive an equation which defines the relation with the distances between the source views and intermediate views and down-sampling ratios.

## Figures and Tables

**Table 1:** Combinations of view synthesis.

No. of Left Views	No. of Synthesized Views	No. of Right Views
37	38	39
37	39	41
39	40	41

**Table 2:** Combination and resolution of down-sampling ratios.

Down-Sampling Ratio (%)	0	20	40	50	75
Champagne_tower	1280 × 960	1024 × 768	768 × 576	640 × 480	320 × 240
Pantomime	1280 × 960	1024 × 768	768 × 576	640 × 480	320 × 240

**Table 3:** PSNR of synthesized views for ClassroomVideo.

Input Views (Down-Sampling Ratio: 50%)	WS-PSNR_Y (dB)	WS-PSNR_U (dB)	WS-PSNR_V (dB)
(1) nearOrg + farDown	31.83	48.90	51.50
(2) nearOrg + farTextureDown	31.49	47.84	50.67
(3) nearDown + farOrg	31.41	48.56	51.16
(4) nearTextureDown + farOrg	31.44	43.74	50.58
(5) nearOrg + farOrg	32.73	49.91	52.49
(6) nearOrg	31.83	48.91	51.50
(7) farOrg	31.43	48.56	51.16

**Table 4:** QPs used for texture and depth.

QP	R1	R2	R3	R4
Texture	22	27	32	37
Depth	17	22	27	32

**Table 5:** Resolution for down-sampling ratio.

Down-Sampling Ratio	0%	12.5%	25%	37.5%	50%
ClassroomVideo	4096 × 2048	3584 × 1792	3072 × 1536	2560 × 1280	2048 × 1024

**Table 6:** Anchor-coded views per class.

Test Class	Sequence Name	No. of Source Views	No. of Anchor-Coded Views	Anchor-Coded Views
A1	ClassroomVideo	15	15	All
A2	ClassroomVideo	15	9	v0, v7, …, v14
A3	ClassroomVideo	15	1	v0

**Table 7:** 3DoF+ test sequence view synthesis frame range.

Test Class	Sequence Name	Frames
A1	ClassroomVideo	89–120
A2	ClassroomVideo	89–120
A3	ClassroomVideo	89–120

**Table 8:** WS-PSNR_Y of synthesized views for different down-sampling ratios.

WS-PSNR_Y (dB)	Regular Output			Masked Output		
Down-Sampling Ratio (%)	ClassroomVideo					
	A1	A2	A3	A1	A2	A3
0	39.46	38.32	29.16	39.34	37.35	26.70
12.5	37.92	37.25	29.10	37.78	36.54	26.86
25	37.33	36.71	29.03	37.21	36.11	26.84
37.5	36.55	36.00	28.90	36.45	35.51	26.83
50	35.30	34.85	28.70	35.22	34.49	26.86

**Table 9:** WS-PSNR_Y of synthesized views for different QPs with A1 class.

WS-PSNR_Y (dB)		A1 (ClassroomVideo)				
	DR ^1^	0%	12.5%	25%	37.5%	50%
QP						
R1		42.08	39.31	38.58	37.59	36.06
R2		40.39	38.62	37.99	37.12	35.74
R3		38.62	37.58	37.04	36.34	35.17
R4		36.77	36.16	35.73	35.16	34.23

^1^ DR represents down-sampling ratio.

**Table 10:** Combination of two down-sampling ratios (DR: down-sampling ratio).

Down-Sampling Combination	DR 1 (%)	DR 2 (%)
D1	0	12.5
D2	0	25
D3	0	37.5
D4	0	50
D5	12.5	25
D6	12.5	37.5
D7	12.5	50
D8	25	37.5
D9	25	50
D10	37.5	50
